# Neuronal stress following exposure to ^56^Fe particles and the effects of antioxidant-rich diets

**DOI:** 10.1093/jrr/rrt155

**Published:** 2014-03

**Authors:** Shibu M. Poulose, Donna Bielinski, Kirsty L. Carrihill-Knoll, Bernard M. Rabin, Barbara Shukitt-Hale

**Affiliations:** 1 Human Nutrition Research Center on Aging, USDA-ARS, Boston, MA 02111, USA; 2 Department of Psychology, UMBC, Baltimore, MD 21250, USA

**Keywords:** neurochemical, autophagy, irradiation, oxidative stress, inflammation

## Abstract

Exposing young rats to particles of high energy and charge (HZE particles), a ground-based model for exposure to cosmic rays, enhances indices of oxidative stress and inflammation and disrupts the functioning of neuronal communication in critical regions of the brain [ 1]. These changes in neuronal function are similar to those seen in ageing [ 2, 3]. Although there is some recovery of function after exposure to ^56^Fe particles, particularly in changes observed 36 h following irradiation, long-term changes (75 days) have been observed, suggesting subcellular damage. Consequently, oxidative stress and inflammation induced by radiation could affect downstream events, such as changes in behavior and gene expression. Therefore, berry fruits high in antioxidant and anti-inflammatory activity, such as blueberries and strawberries, may prevent the occurrence of neurochemical and behavioral changes that occur if fed prior to radiation [ 4].

Rats were exposed to ^56^Fe (1000 MeV/n; 1.5 Gy) particles at the NASA Space Radiation Laboratory at Brookhaven National Laboratory; other rats served as non-irradiated controls. The animals were fed either a control or a 2% blueberry or strawberry diet 8 weeks prior to radiation. Rats were then either euthanized at 36 h (short term) or 30 days following irradiation (long term). Before and after the irradiation, the animals were housed at USDA Human Nutrition Research Center on Aging at Tufts University, Boston.

The results of the experiments indicate that: (1) ^56^Fe exposure caused significant differential, neurochemical changes in critical regions of the brain, such as hippocampus, striatum, frontal cortex and cerebellum, particularly long term. (2) Neurochemical changes resulted in the disruption of autophagy, increased inflammation and increased oxidative stress protein markers. (3) Antioxidant-rich berry diets significantly reduced the accumulation of toxic cellular debris in critical regions of the brain, primarily at the 30 days post-irradiation time-point. (4) Susceptibility to inflammation, autophagy dysregulation, and oxidative stress were proportional to the levels of antioxidant enzymes in the respective brain regions. (5) Exposure to ^56^Fe radiation may cause the accumulation of disease-related proteins such as PHF-Tau, which has been implicated in the pathogenesis of Alzheimer's disease.

Irradiation with ^56^Fe, which causes substantial build-up of toxic cellular debris in critical regions of the brain, may overwhelm the innate antioxidant enzyme defense system [ 5]. Therefore, berry diets high in antioxidants may be used to counter these damaging effects by reducing oxidative stress and inflammation, and activating neuronal housekeeping, in addition to boosting endogenous antioxidant enzymes.

This paper was presented at the NASA Session at Heavy Ion in Therapy and Space Radiation Symposium 2013.

## References

[1] Rabin BM, Shukitt-Hale B, Joseph JA (2007). Relative effectiveness of different particles and energies in disrupting behavioral performance. Radiat Environ Biophys.

[2] Joseph JA, Shukitt-Hale B, McEwen J (2000). CNS-induced deficits of heavy particle irradiation in space: the aging connection. Adv Space Res.

[3] Shukitt-Hale B, Casadesus G, Carey AN (2007). Exposure to ^56^Fe irradiation accelerates normal brain aging and produces deficits in spatial learning and memory. Adv Space Res.

[4] Shukitt-Hale B, Carey AN, Jenkins D (2007). Beneficial effects of fruit extracts on neuronal function and behavior in a rodent model of accelerated aging. Neurobiol Aging.

[5] Poulose SM, Bielinski DB, Carrihill-Knoll K (2011). Exposure to ^16^O particle irradiation causes age-like decrements in rats through increased oxidative stress, inflammation and loss of autophagy. Radiat Res.

